# Novel formulations of oral bisphosphonates in the treatment of osteoporosis

**DOI:** 10.1007/s40520-022-02272-z

**Published:** 2022-11-04

**Authors:** Nicholas Fuggle, Nasser Al-Daghri, Olivier Bock, Jaime Branco, Olivier Bruyère, Enrique Casado, Etienne Cavalier, Bernard Cortet, Maarten de Wit, Andrea Giusti, Philippe Halbout, Nicholas C. Harvey, Mickaël Hiligsmann, Jean-Marc Kaufman, Andreas Kurth, Stefania Maggi, Radmila Matijevic, Salvatore Minisola, Santiago Palacios, Régis Pierre Radermecker, Friederike Thomasius, Sansin Tuzun, Nicola Veronese, John A. Kanis, Jean-Yves Reginster, René Rizzoli, Cyrus Cooper

**Affiliations:** 1 MRC Lifecourse Epidemiology Centre, University of Southampton, Tremona Road, Southampton, SO16 6YD UK; 2 Chair for Biomarkers of Chronic Diseases, Biochemistry Department, College of Science, King Saud University, Riyadh, Kingdom of Saudi Arabia; 3 Department of Osteoporosis, Inselspital-Bern University Hospital, University of Bern, Bern, Switzerland; 4 International Osteoporosis Foundation, Nyon, Switzerland; 5 Centro Hospitalar de Lisboa Ocidental-Hospital Egas Moniz, CEDOC/NOVA Medical School, Nova University of Lisbon, Lisbon, Portugal; 6 Division of Public Health, Epidemiology and Health Economics, WHO Collaborating Center for Public Health Aspects of Musculo-Skeletal Health and Ageing, University of Liège, Avenue Hippocrate 13, CHU B23, 4000 Liege, Belgium; 7 Department of Rheumatology, University Hospital Parc Taulí, I3PT Research Institute (UAB), Sabadell, Barcelona Spain; 8 Department of Clinical Chemistry, University of Liège, CIRM, CHU Sart-Tilman, 4000 Liège, Belgium; 9 Department of Rheumatology, Univ. Lille, CHU Lille, MABlab ULR 4490, 59000 Lille, France; 10 Department of Medical Humanities, Amsterdam University Medical Centre, Amsterdam, The Netherlands; 11 Metabolic Bone Diseases Unit and Fracture Liaison Service, Rheumatology Unit, Department of Medical Specialties, Local Health Trust 3, Via Missolungi 14, 16147 Genoa, Italy; 12 NIHR Southampton Biomedical Research Centre, University of Southampton and University Hospital Southampton NHS Foundation Trust, Tremona Road, Southampton, UK; 13 Department of Health Services Research, CAPHRI Care and Public Health Research Institute, Maastricht University, Maastricht, The Netherlands; 14 Department of Endocrinology, Ghent University Hospital, 9000 Ghent, Belgium; 15 Department of Orthopaedic and Trauma Surgery, Community Clinics Middle Rhine, Campus Kemperhof, Koblenz, Germany; 16 Institute of Neuroscience, Aging Branch, CNR, Padua, Italy; 17 Faculty of Medicine, Clinic for Orthopedic Surgery and Traumatology, Clinical Center of Vojvodina, University of Novi Sad, Novi Sad, Serbia; 18 Department of Clinical, Internal, Anaesthesiology, and Cardiovascular Sciences, Sapienza University of Rome, 00185 Rome, Italy; 19 The Palacios Institute of Women’s Health, Madrid, Spain; 20 Department of Diabetes, Nutrition and Metabolic Disorders, Clinical Pharmacology, University of Liège, CHU de Liège, Liège, Belgium; 21 Center of Bone Health, Frankfurt, Germany; 22 Department of Physical Medicine and Rehabilitation, Cerrahpaşa Medical Faculty, Istanbul University Cerrahpaşa, Istanbul, Turkey; 23 Department of Internal Medicine, Geriatrics Section, University of Palermo, Palermo, Italy; 24 Mary McKillop Institute for Health Research, Australian Catholic University, Melbourne, Australia; 25 Centre for Metabolic Bone Diseases, University of Sheffield, Sheffield, UK; 26 NIHR Musculoskeletal Biomedical Research Unit, University of Oxford, Oxford, UK

**Keywords:** Osteoporosis, Bisphosphonates, Fragility fracture, Therapy, Risedronate, Alendronate

## Abstract

Oral bisphosphonates are a key intervention in the treatment of osteoporosis and in reducing the risk of fragility fractures. Their use is supported by over 3 decades of evidence; however, patient adherence to oral bisphosphonates remains poor in part due to complex dosing instructions and adverse events, including upper gastrointestinal symptoms. This problem has led to the development of novel oral bisphosphonate formulations. Buffered, effervescent alendronate is dissolved in water and so seeks to reduce upper gastro-intestinal adverse events, and gastro-resistant risedronate aims to reduce the complexity of dosing procedure (e.g. fasting prior to consumption) whilst still maintaining the efficacy of fracture risk reduction. Clinical trials and real-world data have been employed to demonstrate some benefits in terms of reduced upper gastro-intestinal adverse events, adherence, persistence and health economic outcomes. This report describes the result of an ESCEO (European Society for Clinical and Economic Aspects of Osteoporosis and Osteoarthritis) expert working group, which explores where oral bisphosphonates sit in current clinical practice guidelines, review their risk–benefit profile and the consequences of poor adherence before exploring novel oral bisphosphonate formulations and their potential clinical and health economic impact. Further research is required but there are signs that these novel, oral bisphosphonate formulations may lead to improved tolerance of oral bisphosphonates and thus, improved adherence and fracture outcomes.

## Introduction

Osteoporosis is a highly prevalent condition affecting older men and women and predisposing to fragility fractures [1, 2]. Oral bisphosphonates have a rich heritage of use in the treatment of osteoporosis but adherence is affected, in large part, by adverse effects [3]. For this reason, new formulations of oral bisphosphonates including gastro-resistant risedronate and buffered alendronate have been produced to improve patient acceptability and an overview of these is potentially beneficial.

Bisphosphonates are pyrophosphate analogues containing a P–C–P bond. The degree of bisphosphonate potency depends on the side-chain length [4] and they have substantial affinity for hydroxyapatite leading to inhibition osteoclast recruitment, osteoclast activity with a subsequent reduction in bone resorption [5].

In this report we describe where oral bisphosphonates sit in current clinical practice guidelines, review their risk–benefit profile and the consequences of poor adherence before exploring the novel oral bisphosphonate formulations and their potential clinical and health economic impact.

## Current clinical practice guidelines

Clinical practice guidelines are defined as “a statement developed systematically to help practitioners and patients make decisions about appropriate medical services” [6] and include recommendations for particular treatments based on review of the available literature at the time [7].

Using the above definition there are a number of clinical practice guidelines relevant to the use of oral bisphosphonates in the treatment of osteoporosis [8] which highlight three main situations when these therapies can be gainfully employed: a first-line therapy in confirmed osteoporosis, a subsequent treatment after bone-forming therapy, and a subsequent treatment after denosumab.

In the first of these use cases, European guidelines for postmenopausal osteoporosis advocate for treatment of high risk patients defined according to the country-specific 10-year fracture risk as calculated by the Fracture Risk Assessment Tool (FRAX®) (modulated by dual-energy X-ray absorptiometry (DXA), trabecular bone score, glucocorticoid therapy, falls history type 2 diabetes and hip axis length as available) or having sustained a fragility fracture of the spine or hip [5].

The exact usage of ‘high risk’ FRAX® thresholds varies across guidelines with the American Association of Clinical Endocrinology/American College of Endocrinology 2020 guidelines for postmenopausal osteoporosis recommending a threshold of 10 years major osteoporotic fracture risk of ≥ 20% or hip fracture risk of ≥ 3% [9].

Those patients at ‘very high risk’, defined as either above the upper assessment threshold on FRAX ® assessment [10, 11], or those with low bone mineral density (BMD) with advanced age, frailty, glucocorticoids, very low T scores or increased falls risk [9] are considered for bone-forming therapies first-line if these are available [9, 10].

In the second use case, anti-resorptive therapies, including oral bisphosphonates are used to ‘lock-in’ the benefits accrued from bone-forming therapies [12] and in the third case, they are used similarly to reduce the immediate, rebound fracture risk which occurs after discontinuing denosumab [5, 13].

In the majority of the cases above, an oral bisphosphonate should be used as first-line, with intravenous preparations and denosumab as alternatives if oral bisphosphonates are contraindicated or not tolerated [5, 9]. Treatment with oral bisphosphonates should be reviewed every 3–5 years [5] with the AACE/ACE recommending additional years (up to 5 years) of treatment to be considered if the fracture risk remains high at assessment [9].

Oral bisphosphonates form a cornerstone of osteoporosis therapy and their benefits are established; however, their risks need to be addressed, particularly when consenting patients for therapy.

## Risk–benefit

Oral bisphosphonates have been available for the treatment of osteoporosis since the release of etidronate in the early 1990s [14, 15], alendronate in the mid-1990s [16, 17] and risedronate in the late-1990s [18–20] with risk reduction for radiographic vertebral fractures of 40–50% at 3–5 years compared to placebo and non-vertebral fracture risk reduction of 20–30%.

Oral bisphosphonates can be associated with mild, common adverse effects, including upper gastro-intestinal irritation which will be addressed later as they are relevant to the novel bisphosphate formulations; however, there are important, rare adverse events which require particular attention as they often arise in consultations when consenting a patient in clinic. These include osteonecrosis of the jaw, atypical femoral fractures and cardiovascular effects.

Osteonecrosis of the jaw is a rare condition defined by the presence of exposed, necrotic bone lesions which are present beyond 8 weeks and do not respond to appropriate therapy [21]. It occurs mainly in the mandible but the maxilla can also be affected and may occur in response to potent anti-resorptive therapy with the diagnosis excluded by the use of previous radiotherapy to the affected area. It is associated with poor oral hygiene, smoking, diabetes, concomitant glucocorticoid or chemotherapy, and invasive dental procedures (including dental extractions or implants) but even so, it has a small incidence rate of 1–90/100,000 patient-years [22, 23].

Atypical femoral fractures occur in the subtrochanteric and diaphyseal region of the femur and are associated with bisphosphonate usage [24]. The absolute risk of occurrence is low with incidence rates of 1.8/100,000 patient-years after 2 years of therapy, though the risk does rise with increasing length of treatment leading to 113/100,000 patient-years at 8–9.9 years of exposure to bisphosphonates [22, 24–26]. The risk of atypical fractures is outweighed by the benefit of anti-resorptive therapy as a study of 1.8 million patients demonstrated that, over a 5-year period, 162 fragility fractures of the spine, hip and forearm are saved for each atypical femoral fracture sustained [26].

The cardiovascular effects of bisphosphonates are debated, with data from animal studies (using higher dosages than used in human studies) suggesting potential cardiovascular protective effects due to reduced atherosclerosis in response to bisphosphonate therapy [27–30]. This is supported by a limited signal from a trial of risedronate which showed a protective effect of 2.5 mg dose for cardiovascular mortality (RR 0.69, 95% CI 0.49–0.99) and stroke mortality (RR 0.36, 95% CI 0.17–0.78), though this effect is caveated by the fact that there was no significant effect at the 5 mg dose or on the incidence of coronary artery disease [31]. Indeed, meta-analyses have shown no significant associations (either protective or adverse) between bisphosphonates and cardiovascular death, adverse cardiovascular outcomes, myocardial infarction or stroke [32] and this is echoed by long term, prospective database studies [33].

## Consequences of poor adherence

The adherence to a medication is defined as the process by which patients take a medication. This can be divided into three phases; initiation, implementation and persistence [34, 35] and can be measured, for example, by the medication possession ratio (MPR, the number of days covered by treatment in a given period of time divided by the total number of days in that period) [36]. Forms of ‘non-adherence’ can, therefore, include non-initiation of treatment, errors in dosing (including missed doses or taking the medication incorrectly) and discontinuing the treatment [34, 35].

In terms of initiation, a study of over 75,000 patients found that between 20 and 30% did not initiate a prescribed medication [37]. This is particularly concerning given the established osteoporosis ‘Treatment Gap’ [38] with a Belgian study demonstrating that of over 23,000 patients sustaining a hip fracture, only 6% were commenced on anti-osteoporosis treatment within 1 year [39].

When considering the implementation phase, it is important to note that the majority of established bisphosphonates are ‘immediate-release’ formulations which have approximately 1% bioavailability as their absorption is limited by chelation with aluminium, magnesium and calcium cations [40]. For this reason, these immediate-release preparations require complex dosing instructions including taking them on a fasted stomach (to avoid chelation by foods containing the above cations), with water and up to 2 h of fasting after consumption (thus delaying breakfast), with strict instructions to remain upright (and not lie down) after consumption [41] and to be taken separately from other medications. These complexities affect adherence with an estimated 50% of patients not following the above recommendations [42].

A survey of 36 women aged ≥ 55 years taking immediate-release bisphosphonates found that 56.5% were non-adherence with dosing instructions, and further, that 40.5% were non-adherent with instructions related to improving absorption (e.g. fasting prior to dosing) and 34.7% were non-adherent with instructions related to adverse effects (e.g. remaining upright following dosing) [42].

Discontinuation of medication is common with rates up to 40% by 12 months for all medications [43] and as high as 85% by 3 years with anti-osteoporosis medications [39]. A systematic review of 89 real-world studies revealed a 12 months persistence range of 17.7—74.8% for oral bisphosphonates with a mean MPR of 28.2—84.5% over the same time period [44]. The determinants identified from this study included geographic residence, marital status, tobacco use, educational status, income, hospitalisation, medication type and dosing frequency [44]. A further systematic review of 10 studies specifically investigating adherence and persistence to bisphosphonate therapy in postmenopausal women and found a mean MPR range of 54–71% at 1 year and persistence ranged from 28 to 74% [45]. Though these ranges are large, the authors did note that adherence and persistence were higher in formulations which required less frequent dosing and fracture rates were significantly lower in women with higher adherence (particularly a MPR > 80%) [45]. This finding is supported by data from national claims database which found that the MPR of bisphosphates rose with increasing interval between prescribed doses (from daily to 3 monthly), though some of the included medications were intravenously delivered [46].

This poor adherence leads to suboptimal efficacy in terms of fracture risk reduction [47–49] and an increased financial burden to the health system [50, 51]. Healthcare professionals play a vital role in ensuring that correct dosing instructions are provided and, wherever possible, adhered to. Indeed, adherence can be monitored using bone turnover markers [52].

The determinants of poor adherence are manifold, and social (e.g. low health literacy), economic (e.g. lack of healthcare insurance), healthcare system (e.g. healthcare provider communication skills), condition-specific (e.g. the lack of symptoms of osteoporosis), therapy-specific (e.g. the lack of immediate, tangible medication benefit) and patient-related factors (e.g. visual impairment) all play a role [53].

Data from the ‘Screening in the community to reduce fractures in older women’ (SCOOP) trial showed that the FRAX® input characteristics which were significantly associated with improved adherence over 5 years of follow-up included younger age (OR 0.96, *p* = 0.01), parental fractured hip (OR 1.67, *p* < 0.01) and having a dual-energy X-ray absorptiometry (DXA) scan (OR 1.89, *p* < 0.01) [54]. Importantly, those in a higher FRAX® risk category were also significantly more adherent to anti-osteoporosis medications (OR 2.80, *p* = 0.02) [54].

Interventions which have strong evidence to support their usage in improving medication adherence (in osteoporosis and other conditions) include patient counselling, patient education in combination with counselling, adherence monitoring combined with counselling, reminders to take medication and dose simplification including flexibility of dosing regimens [53]. From a fairly broad literature, this represents a small proportion of possible interventions with the majority showing little or no improvement [53], but it does appear that multicomponent interventions which incorporate patient education have the most marked beneficial effects [55].

The key steps to improving adherence include understanding the problem at hand (initiation, implementation or persistence), accurately measuring adherence and robustly identifying the reasons for non-adherence so that they can be addressed [53]. In addition, there have been therapeutic developments with novel oral bisphosphonates, which aim to reduce the therapy-specific factors impairing adherence.

## Novel formulations

Novel formulations which overcome the complexities of drug delivery have been developed including buffered alendronate and gastro-resistant risedronate.

### Buffered alendronate

Upper gastro-intestinal adverse effects are a substantial adverse effect when using bisphosphonates for the treatment of osteoporosis [56]. Indeed, in an acidic environment (with a pH < 3) alendronate is in a free acidic form which is far more irritant to the gastro-oesophageal mucosa than the sodium salt (alendronate sodium) form [57].

A possible solution for the problem is novel, effervescent alendronate which aims to improve tolerability by dissolving the active alendronate in a buffered solution with pH 4.8–5.4 (taken in at least 120 ml of water) [58] to reduce gastro-oesophageal irritation.

A prospective, observational, post-authorisation study in Italy and Spain examined the performance of a form of buffered alendronate (Binosto®) focusing on key outcomes of adverse events, medication errors, persistence and compliance, in a group of post-menopausal females (intention to treat *n* = 1084, safety cohort *n* = 1028, completed *n* = 873) over a 12-month period [59].

The mean age of participants was 67 years and just over a third (36%) had sustained a fracture previously. History of Gastrointestinal symptoms were observed in 31% (*n* = 319) participants and, of these, 85% (*n* = 271) were upper gastrointestinal. The cumulative incidence of all upper gastro-intestinal adverse events was 12.7%, with 9.6% specifically related to the effervescent, buffered alendronate (8% mild, 1.5% moderate, 0.2% severe but none of these adverse events were graded as serious) [59].

In terms of the specific symptoms or pathologies associated with these adverse effects the incidence rate was highest for nausea (IR = 2.1, 95% CI 1.4–3.2) followed by abdominal pain (IR = 1.2, 95% CI 0.6–2.1) and gastritis (IR = 0.8, 95% CI 0.4–1.6) with no reported gastric stenosis, ulceration, perforation or haemorrhage [59].

Medication errors were recorded in 30% of participants (*n* = 307) including not waiting a sufficient time before consuming the first meal (10%), using a liquid other than water to dissolve the effervescent alendronate (9%) and an insufficient volume of water (< 30 ml) drunk following the consumption of the medication (7.6%) [59].

Approximately 80% of participants continued the effervescent alendronate through the duration of follow-up with the most common cause of discontinuation labelled as “patient decision” in 42.6% (*n* = 89) of those who discontinued [59]. Persistence rates were therefore higher than that reported in bisphosphonates in a similar study in which 71.3% participants were taking a bisphosphonate at 6 months of follow-up.

The percentage of upper gastro-intestinal adverse events related to the effervescent alendronate (9.6%) was lower than those reported in studies of alendronate in populations of post-menopausal women which include 30% in the Fracture Intervention Trial [60] and above 20% in the Fosamax International Trial Study [61] and other early studies of alendronate [62, 63].

Real-world data on effervescent alendronate comes from a clinical database study by Giusti and colleagues, which recruited postmenopausal women with osteoporosis (T ≤ − 2.5 or T ≤ 2.0 with a vertebral fracture) comparing effervescent alendronate (*n* = 144) to conventional alendronate (*n* = 216) [64]. Persistence was measured and was found to be significantly higher in the effervescent group at 6 months (91 vs 75%, *p* < 0.01) and 12 months (81 vs 69%, *p* = 0.009). Discontinuation due to upper gastro-intestinal adverse events was significantly lower in the effervescent group (4 vs 11%, *p* = 0.027) as was discontinuation due to patient decision (6 vs 13%, *p* = 0.016). The findings of this study are from a single area in North–West Italy and did not include adherence data; however, they suggest that the real-world experience of effervescent alendronate may be favourable for upper gastro-intestinal adverse events and persistence rates.

One of the current issues regarding the widespread usage of effervescent alendronate is that it is currently available in a relatively small number of countries, though work is ongoing to broaden the geographic scope of supply.

### Gastro-resistant risedronate

Gastro-resistant risedronate is coated with a gastro-resistant, ethylenediaminetetraacetic acid (EDTA) coating which acts as a food stabiliser and calcium chelator and means that it bypasses the oesophagus and stomach [41] prior to tablet disintegration more distally in the gastro-intestinal tract [65]. This eliminates the need for pre-consumption fasting and therefore allows more ‘user-friendly’ instructions for drug consumption and provides potentially better drug absorption regardless of prior food intake (or fasting status) [41, 66].

Gastro-resistant risedronate taken before (*n* = 308) and after (*n* = 307) breakfast was compared to immediate-release risedronate (*n* = 307) and was found to perform similarly in all three groups with regard to efficacy (percentage change in lumbar spine BMD) and safety outcomes and approximately 80% of participants completed the initial 12 months of a non-inferiority study [67]. When this study was extended to 2 years comparable increases in BMD were observed across all three groups but significantly greater CTX decreases were observed in the gastro-resistant treatment cohort [66]. There were no significant differences in incident vertebral fractures (gastro-resistant risedronate before breakfast = 6, gastro-resistant risedronate after breakfast = 2 and immediate-release risedronate = 2). Medication completion rates were similar (gastro-resistant risedronate before breakfast 76.2%, gastro-resistant risedronate after breakfast 77.2% and immediate-release risedronate 80.8%) but rates of upper abdominal pain adverse events were higher in the gastro-resistant risedronate before breakfast group (gastro-resistant risedronate before breakfast 7.5%, gastro-resistant risedronate after breakfast 2.9% and immediate-release risedronate 2.3%).

Beyond randomized controlled trials, real-world studies have provided insights regarding safety and efficacy. A study of women in the IBM MarketScan Commercial and Medicine Supplemental databases (spanning 10 years from 2009 to 2019) used a retrospective, observational design to compare gastro-resistant risedronate to all other oral bisphosphonates [36]. Patients were matched for ages, year of index bisphosphonate administration, insurance plan type, region, comorbidity index, fracture and use of drugs affecting BMD.

Drug adherence was measured with persistence defined as the time from initiation to the end of treatment, discontinuation was defined as a treatment gap of ≥ 90 consecutive days and adherence was measured according to the MPR.

Over more than 50 months of observation, 16,737 women took gastro-resistant risedronate and 1,348,153 took other oral bisphosphonates (66.3% alendronate, 20.2% ibandronate, 13.5% immediate-release risedronate) leading to matched groups containing 2726 patients with a median age of 60.0 years and 1.7% were diagnosed with a fracture during the baseline study period.

The prescription of gastro-resistant risedronate led to a 17% reduction in overall risk of fracture (incidence rates per 1000 patient-years 34.65 on gastro-resistant risedronate vs 42.13 on other oral bisphosphonates, *p* < 0.05) and a 29% reduction in the risk of spinal fracture (incidence rates per 1000 patient-years 10.84 on gastro-resistant risedronate vs 15.13 on other oral bisphosphonates, *p* < 0.05). This benefit in fracture rate was seen numerically over time, though was only significant at 36 months (7.08% on gastro-resistant risedronate vs 8.67% on other oral bisphosphonates, *p* < 0.05).

When compared to the most common oral bisphosphonate (alendronate), those initiated on gastro-resistant risedronate had a 19% lower risk of any fracture and a 31% lower risk of spinal fracture.

Persistence on treatment was generally poor with the majority of patients discontinuing their index treatment within 2 years of initiation (80.5% discontinuation rate of gastro-resistant risedronate, 74.4% discontinuation rate of other bisphosphonates over 2 years) though persistence was lower for gastro-resistant risedronate particularly due to high rates of discontinuation within the first months of commencing therapy. These results are within the spectrum of mean persistence for bisphosphonates recorded from prospective and retrospective observational studies with 2 years mean persistence rates of 12.9–60.6% and mean compliance of 34.5–47.9% over the same time period [44].

In a further US claims database analysis comparing gastro-resistant risedronate with immediate-release risedronate and alendronate, the gastro-resistant preparation was associated with a lower rate of fracture in women with osteoporosis [68]. This was observed in subgroup analyses for those aged ≥ 65 years (incidence risk ratio (IRR) 0.63 (95% CI 0.46–0.86) for any fracture, IRR 0.41 (95% CI 0.18–0.93) for pelvic fracture), those aged ≥ 70 years (IRR 0.63 (95% CI 0.50–0.96) for any fracture, IRR 0.24 (95% CI 0.08–0.68) for pelvic fracture) and in a ‘high-risk’ population with older age, comorbidity and using medications which increase fracture risk (IRR 0.36 (95% CI 0.16–0.82) for pelvic fracture). In this study, the discontinuation rate for bisphosphonates was 40% within 1 year. Although limited by a young population (due to the nature of the database) and the use of a coded diagnoses, this study suggests potential benefits, in terms of fracture protection, of gastro-resistant risedronate compared to commonly used immediate-release preparations.

## Economic benefits of new formulations

Both buffered, effervescent alendronate and gastro-resistant risedronate have the potential to improve adherence which can lead to reduced fractures and improved health outcomes and reduced costs associated with fractures. However, these novel preparations are more expensive than the standard formulations which will increase costs, and increased adherence will also lead to an increase in drug costs.

The economic value of a drug is typically assessed using a cost-effectiveness analysis which aims to answer the question “Is the intervention worth the price?”. The result of a cost-effectiveness analysis is expressed using the incremental cost effectiveness ratio (ICER), a measure of the difference between an intervention and comparator calculated as the total cost divided by the difference between the intervention and comparator in terms of Quality Adjusted Life Years (QALY, equivalent to 1 year lived in perfect health). The ICER is measured against a threshold for cost–effectiveness, which can be estimated as a multiple of GDP per capita.

When considering the wider utility of these novel formations of bisphosphonates health economic analyses should be conducted in line with the ESCEO-IOF recommendations [69] to ensure that decision-makers and policy-makers are adequately informed of the economic impact of introducing these novel formulations so that the most cost-effective interventions can be identified and healthcare resources allotted accordingly.

The cost-effectiveness of effervescent alendronate was assessed using a Markov microsimulation model adjusted to the Italian healthcare system (using fracture data from 2017 discharge summaries) and incorporating the costs and consequences of fracture events over a lifetime of women up to the age of 100 years (or death) [70]. Effervescent alendronate was compared to alendronate, denosumab, zoledronate and no treatment. Participants were aged 60–80 years and had T-scores ≤ − 3.0 or an existing vertebral fracture. Persistence data provided 12 months of follow-up with the model extended to 3 years to better reflect clinical practice [70].

The economic analysis demonstrated that effervescent alendronate was cost-effective compared to all comparators for postmenopausal women ≥ 60 years in Italy. In subgroup analyses, effervescent alendronate was dominant (indicating more QALYs for lower total costs) for cost-effectiveness over denosumab at 1 and 3 years for both low BMD and vertebral fracture groups, over alendronate at 1 and 3 years for the low BMD group ≥ 65 years and the fracture group ≥ 75 years and cost-saving compared to no treatment at 1 and 3 years for the low BMD group ≥ 65 years and the fracture group ≥ 75 years. Effervescent alendronate was dominant over zoledronate at 3 years but not at 1 year as it was assumed that persistence for zoledronate over 1 year would be 100% [70]. However, even at 1 year the ICER was below € 20,000 (€ 2019) per QALY. The persistence data for this Italian model was taken from the 12-month study by Giusti and colleagues [64] and extrapolated to 3 years which could be a limitation, however, the model conservatively assumed similar rates of adverse effects from effervescent alendronate compared to conventional alendronate.

Similar Markov model, microsimulation methods were used for a study of the cost-effectiveness of gastro-resistant risedronate in a French population of postmenopausal women aged 60–80 years with BMD T-scores ≤ − 2.5 with or without vertebral fractures [47]. In this case, efficacy data were taken from a previous meta-analysis and persistence data drawn from an Australian longitudinal study (as no French data for persistence on gastro-resistant risedronate were available).

This found that gastro-resistance risedronate was cost-effective compared with alendronate, generic risedronate and no treatment, in a French population of postmenopausal women [47]. Cost-effectiveness improved with increasing age and with increasing risk of fracture at baseline.

In subgroup analyses, gastro-resistant risedronate was dominant over immediate-release risedronate and alendronate in those ≥ 75 years and cost saving compared to no treatment in those ≥ 70 years [47].

The cost-effectiveness of gastro-resistant risedronate is supported by the findings of a US, healthcare resource utilization study of claims data which demonstrated a significantly lower number of hospital in-patient stays for patients on gastro-resistant risedronate compared to other oral bisphosphonates (IRR 0.86, 95% CI 0.76–0.97) and subsequently lower costs for in-patient healthcare utilization ($993 per patient per year lower, *p* < 0.05) [36]. However, there was no significant difference for emergency department, out-patient or ambulatory costs and pharmacy costs were higher for gastro-resistant risedronate compared to other oral bisphosphonates ($680 per patient per year higher). Indeed costs, in particular out-of-pocket costs, were considered to be a likely cause for patient discontinuation of gastro-resistant risedronate, as the mean cost of a 28–30-day supply was $ 42.39 for gastro-resistant risedronate compared to $10.72 for other bisphosphonates [36].

These economic analyses highlight the potential health economic benefits of novel bisphosphonate formulations; however, further work in other populations would assist in supporting these findings.

## Conclusions

Oral bisphosphonates play a key role in the treatment of osteoporosis and the amelioration of fracture risk. They are, however, hampered by adverse events, particularly affecting the upper gastro-intestinal tract, which reduce patient adherence and persistence. Effervescent alendronate and gastro-resistant risedronate seek to improve adherence by simplifying the complex dosing rigmarole and reducing upper gastro-intestinal adverse events. Data from trials and real-world data are encouraging with regard to the benefits of these medications both clinically and from a health economic perspective. Further research across diverse populations and focusing on fracture outcomes will strengthen the body of evidence around these formulations.

## Declarations

### Content to participants

The choice of topics, participants, content and agenda of the Working Groups as well as the writing, editing, submission and reviewing of the manuscript are the sole responsibility of the ESCEO, without any influence from third parties.
