# Correction: Novel formulations of oral bisphosphonates in the treatment of osteoporosis

**DOI:** 10.1007/s40520-022-02319-1

**Published:** 2023-01-03

**Authors:** Nicholas Fuggle, Nasser Al-Daghri, Olivier Bock, Jaime Branco, Olivier Bruyère, Enrique Casado, Etienne Cavalier, Bernard Cortet, Maarten de Wit, Andrea Giusti, Philippe Halbout, Nicholas C. Harvey, Mickaël Hiligsmann, Jean-Marc Kaufman, Andreas Kurth, Stefania Maggi, Radmila Matijevic, Salvatore Minisola, Santiago Palacios, Régis Pierre Radermecker, Friederike Thomasius, Sansin Tuzun, Nicola Veronese, John A. Kanis, Jean-Yves Reginster, René Rizzoli, Cyrus Cooper

**Affiliations:** 1 MRC Lifecourse Epidemiology Centre, University of Southampton, Tremona Road, Southampton, SO16 6YD UK; 2 Chair for Biomarkers of Chronic Diseases, Biochemistry Department, College of Science, King Saud University, Riyadh, Kingdom of Saudi Arabia; 3 Department of Osteoporosis, Inselspital-Bern University Hospital, University of Bern, Bern, Switzerland; 4 International Osteoporosis Foundation, Nyon, Switzerland; 5 Centro Hospitalar de Lisboa Ocidental-Hospital Egas Moniz, CEDOC/NOVA Medical School, Nova University of Lisbon, Lisbon, Portugal; 6 Division of Public Health, Epidemiology and Health Economics, WHO Collaborating Center for Public Health Aspects of Musculo-Skeletal Health and Ageing, University of Liège, Avenue Hippocrate 13, CHU B23, 4000 Liege, Belgium; 7 Department of Rheumatology, University Hospital Parc Taulí, I3PT Research Institute (UAB), Sabadell, Barcelona Spain; 8 Department of Clinical Chemistry, University of Liège, CIRM, CHU Sart-Tilman, 4000 Liège, Belgium; 9 Department of Rheumatology, Univ. Lille, CHU Lille, MABlab ULR 4490, 59000 Lille, France; 10 Department of Medical Humanities, Amsterdam University Medical Centre, Amsterdam, The Netherlands; 11 Metabolic Bone Diseases Unit and Fracture Liaison Service, Rheumatology Unit, Department of Medical Specialties, Local Health Trust 3, Via Missolungi 14, 16147 Genoa, Italy; 12 NIHR Southampton Biomedical Research Centre, University of Southampton and University Hospital Southampton NHS Foundation Trust, Tremona Road, Southampton, UK; 13 Department of Health Services Research, CAPHRI Care and Public Health Research Institute, Maastricht University, Maastricht, The Netherlands; 14 Department of Endocrinology, Ghent University Hospital, 9000 Ghent, Belgium; 15 Department of Orthopaedic and Trauma Surgery, Community Clinics Middle Rhine, Campus Kemperhof, Koblenz, Germany; 16 Institute of Neuroscience, Aging Branch, CNR, Padua, Italy; 17 Faculty of Medicine, Clinic for Orthopedic Surgery and Traumatology, Clinical Center of Vojvodina, University of Novi Sad, Novi Sad, Serbia; 18 Department of Clinical, Internal, Anaesthesiology, and Cardiovascular Sciences, Sapienza University of Rome, 00185 Rome, Italy; 19 The Palacios Institute of Women’s Health, Madrid, Spain; 20 Department of Diabetes, Nutrition and Metabolic Disorders, Clinical Pharmacology, University of Liège, CHU de Liège, Liège, Belgium; 21 Center of Bone Health, Frankfurt, Germany; 22 Department of Physical Medicine and Rehabilitation, Cerrahpaşa Medical Faculty, Istanbul University Cerrahpaşa, Istanbul, Turkey; 23 Department of Internal Medicine, Geriatrics Section, University of Palermo, Palermo, Italy; 24 Mary McKillop Institute for Health Research, Australian Catholic University, Melbourne, Australia; 25 Centre for Metabolic Bone Diseases, University of Sheffield, Sheffield, UK; 26 NIHR Musculoskeletal Biomedical Research Unit, University of Oxford, Oxford, UK

**Correction: Aging Clinical and Experimental Research (2022) 34:2625–2634** 10.1007/s40520-022-02272-z

In the published article, the author would like to update the following lines from

Gastrointestinal adverse events were observed in 31% (*n* = 319) participants and, of these, 85% (*n* = 271) were upper gastrointestinal.

to

History of Gastrointestinal symptoms were observed in 31% (*n* = 319) participants and, of these, 85% (*n* = 271) were upper gastrointestinal

in the published article.

The original article has been updated.

